# Ventricular tachycardia during microdebrider-assisted turbinectomy: An electrocardiographic artifact

**DOI:** 10.4103/1658-354X.65124

**Published:** 2010

**Authors:** Khalid Sofi, Ab Salam Khalid, Kurdi A. Mushtaq Gilani

**Affiliations:** *Department of Anesthesia, King Abdul-Aziz Medical City, King Fahad National Guard Hospital, Riyadh, KSA*

**Keywords:** *Turbinectomy*, *microdebrider*, *ventricular tachycardia*

## Abstract

Interference of monitored electrocardiogram is common during different surgical procedures using electrical equipment. The electrical devices used induce artifacts in the electrocardiographic tracing, which may resemble serious arrhythmia. We describe a case of electrocardiographic artifact resembling ventricular tachycardia with the use of a Storz unidrive microdebrider during inferior turbinectomy under general anesthesia. This case report highlights the importance of knowledge of various equipment-related electrocardiographic artifacts in avoiding unnecessary and harmful therapeutic interventions.

## INTRODUCTION

Turbinectomy is a commonly performed ENT procedure, mostly in healthy patients. The procedure is performed under general anesthesia with standard monitoring which includes non-invasive BP (NIBP), pulse oximetry and 5-lead electrocardiography. Electrical interference of the displayed EKG is very common from a wide variety of equipments used by the surgeons. We encountered one such case in EKG interference from a Storz Microdebrider, resembling the alarming ventricular tachycardia (VT). We present this case for the clinicians to avoid unnecessary and harmful therapeutic interventions. A similar case has been reported by Gaiser *et al*. during functional endoscopic sinus surgery (FESS) with the use of Xomed straight shot microdebrider.[1]

## CASE REPORT

**Figure 1:**
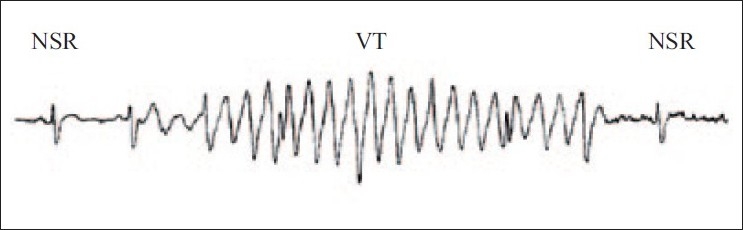
ECG artifact resembling VT during the use of microdebrider; NSR, normal sinus rhythm; VT, ventricular tachycardia

A 29-year-old female with a body mass index (BMI) of 24.2 kg/m^2^ and no past exposure to anesthesia was admitted for septoplasty and inferior turbinectomy as a day case. The patient was examined in the anesthesia clinic and cleared for the procedure as ASA I. The patient was reassessed before the procedure and premedicated with midazolam 1.5 mg intravenously. Standard anesthesia monitoring with NIBP, pulse oximetry and 5-lead EKG was started. Pre-induction vital signs were BP 118/65 mmHg, pulse 69 beats/minute, oxygen saturation (SpO_2_) 99% on room air and EKG showed a sinus rhythm. Anesthesia was induced with propofol, fentanyl and cisatracurium followed by orotracheal intubation with size 7 RAE tube. Patient was put on pressure-controlled ventilation using Drager Zeus Anesthesia machine and anesthesia was maintained with desflurane and oxygen:air mixture. Intraoperative systolic BP stayed around 100 mmHg. The surgeon completed septoplasty in an hour without any problems and started inferior turbinectomy using Storz unidrive microdebrider. As soon as the surgeon used the microdebrider, EKG waveform resembled VT which lasted for 2–3 s when the surgeon was asked to stop [Figure 1]. During this period the patient had a good pulse and regular waveform on pulse oximeter inconsistent with VT. A possible faulty current from the microdebrider was the reason for EKG artifact mimicking VT. After checking the leads and connections, debrider was used again and a similar EKG waveform resembling VT returned on the monitor. The patient was absolutely stable with a BP of 100/60 mmHg, heart rate of 65 beats/minute, SpO_2_ of 100% and end-tidal CO_2_ of 33 mmHg. Another microdebrider, Meditron Straight Shot was connected and the procedure started. The procedure was completed without any EKG interference. Patient was extubated, shifted to post anesthesia care unit and remained in sinus rhythm throughout. The Storz Microdebrider was handed over to Biomedical Engineering for necessary repairs and was found to have some loose connections which were tightened and the problem was corrected. The actual rhythm strip of EKG artifact could not be produced due to yet to be installed software for data retrieval, in the new Zeus anesthesia machine from Drager in our department [Figure 1].

## DISCUSSION

Intraoperative monitoring, identification of critical incidents and institution of appropriate therapy are the sole responsibilities of the anesthesiologist. All recommending bodies around the globe consider EKG monitoring as part of standard basic anesthesia monitoring and apply its use to procedures under general anesthesia, regional anesthesia and conscious sedation.[2] The EKG has proven to be a valuable monitor of the patient during surgery, being inexpensive and easy to use. However, interference of the displayed EKG from a wide variety of sources is still common intraoperatively. Any electrical activity on the EKG that is noncardiac in origin is an artifact and may result from patient tremor or movement, loose electrodes, broken cables or broken wires of the monitor. Another important source of EKG artifact is the interference from electrical equipment used intraoperatively. Pressure-controlled irrigation pump (during arthroscopy)[3] deep brain stimulator[4] and microdebrider are some of the common equipments associated with EKG artifacts.

Since its introduction in 1993, microdebrider is gaining popularity for use in a wide variety of otorhinolaryngology, and head and neck surgical procedures. They are commonly used in endoscopic sinus surgery, turbinoplasties,[5,6] rhinoplasties, treatment of choanal atresia, adenoidal hypertrophy, septoplasties and removal of tumors.

In our case, a Storz Unidrive microdebrider resulted in an EKG artifact resembling VT, the source of interference being a stray current from loose electrical wiring of the microdebrider. All electrical equipments used intraoperatively are supposed to be grounded to allow any stray electric current to be carried of the ground. However, a malfunction of the equipment results in failure of the grounding of the current as was seen in this case.

VT is defined as three or more consecutive ventricular premature beats. The usual rate is 160–240 beats/minute. Treatment for this rhythm is cardioversion and/or pharmologic therapy with amiodarone or lidocaine depending on the hemodynamic compromise.[7] VT is usually easy to diagnose during intraoperative EKG monitoring. Patients with implantable cardiac defibrillator (ICD) or a pacemaker are at risk of the device malfunction as a result of electrical interference. The stray electric current can cause the ICD to discharge or prevent a pacemaker from firing.[8] Hence, other monitors of cardiac rhythm such as pulse oximeter or palpation of the pulse are invaluable in these patients intraoperatively.

Any electrical equipment is liable to develop stray currents through loose connections and results in interference of monitored EKG. An improved understanding of the artifacts generated by equipment and their identifying characteristics is important to avoid misinterpretation, misdiagnosis and iatrogenic complications.[9] Our case is similar to the one reported by Gaiser *et al*. and alerts the clinicians, in particular anesthesiologists, to have proper understanding of the electrocardiographic artifacts during various surgical procedures. Misdiagnosis of electrocardiographic artifact as VT may lead to unnecessary interventions.[10]

This case report aims to let the anesthesia community know about the various equipments which can generate abnormal benign EKG artifacts to, avoid unnecessary intervention and harm to the patient.
